# Early hemodynamic changes following endoscopic sclerotherapy for hemorrhoidal disease assessed by transperineal ultrasound and their relationship with clinical outcomes

**DOI:** 10.1007/s00384-026-05165-z

**Published:** 2026-06-08

**Authors:** Gianpiero Gravante, Veronica De Simone, Arcangelo Picciariello, Francesco Palmieri, Claudio Missaglia, Roberto Sorge, Salvatore Sorrenti, Pierpaolo Sileri, Gaetano Gallo

**Affiliations:** 1 Department of General Surgery, Azienda Sanitaria Locale ASL Lecce, Casarano, Italy; 2 Colorectal Surgery Unit, IRCCS San Raffaele Scientific Institute, Faculty of Medicine and Surgery, Vita-Salute University, Via Olgettina 60, 20132 Milan, Italy; 3 Department of Experimental Medicine, University of Salento, Lecce, Italy; 4 Department of Human Physiology, Laboratory of Biometry, University of Tor Vergata in Rome, Rome, Italy; 5 Department of Surgery, “Sapienza” University of Rome, Rome, Italy

**Keywords:** Pelvic floor diseases, Proctology, Doppler imaging

## Abstract

**Background:**

Polidocanol foam (PF) sclerotherapy has regained interest as a minimally invasive treatment for hemorrhoidal disease (HD). However, the early hemodynamic effects of sclerotherapy and their relationship with clinical outcomes remain poorly defined. This study aimed to evaluate early local hemodynamic changes following endoscopic PF sclerotherapy using transperineal ultrasound (TPUS) and to explore their association with patient-reported outcome measures (PROMs).

**Methods:**

This prospective observational study included patients with Goligher grade I–IV HD treated with endoscopic PF sclerotherapy. TPUS Doppler assessment of peak systolic velocity (PSV), end-diastolic velocity (EDV), and resistance index (RI) was performed at baseline, 7 days, and 30 days post-treatment. Symptoms were evaluated using the PROM-HISS score. Hemodynamic and clinical outcomes were compared over time and stratified by HD severity (Goligher I–II vs III–IV).

**Results:**

Thirty-seven patients completed follow-up. No significant differences in preoperative PSV were observed between lower- (Goligher I/II) and higher-grade HD (Goligher III/IV). After treatment, patients with lower-grade HD showed a significant reduction in PSV and RI at both 7 and 30 days (*p* < 0.01), indicating effective modulation of arterial inflow. In contrast, no significant changes in PSV or RI were observed in higher-grade HD, while EDV increased at 30 days (*p* = 0.012). PROM-HISS scores significantly improved in all patients at 7 days; however, symptom scores increased between 7 and 30 days in higher-grade HD.

**Conclusions:**

PF sclerotherapy induces early short-term hemodynamic changes detectable by TPUS in lower-grade HD, paralleling consistent short-term symptom improvement. In advanced HD, clinical benefit appears transient and not supported by objective vascular remodeling. TPUS emerges as a valuable non-invasive tool for functional assessment, follow-up, and treatment stratification after sclerotherapy.

## Introduction

Sclerotherapy for the treatment of hemorrhoidal disease (HD) has recently regained attention [1]. The introduction of new formulations of established agents—particularly 3% polidocanol foam (PF)—has significantly improved the safety profile of the procedure and progressively replaced phenol oil as the preferred sclerosant [2–4]. Clinical studies involving patients with Goligher grade II–III hemorrhoids, as well as selected high-risk populations, have demonstrated favorable efficacy and safety profiles [5–8], with preliminary but encouraging long-term outcomes also reported [9]. Recurrences can be effectively managed, as the procedure is office-based, minimally symptomatic, and easily repeatable [10]. Importantly, current evidence suggests that the indication for sclerotherapy should primarily be guided by symptom burden rather than by Goligher classification alone, as HD is increasingly recognized as a symptomatic condition with a limited correlation with anatomical grading [11]. On this basis, sclerotherapy may be considered a first-line treatment for lower-grade HD and a reasonable palliative option for patients with advanced disease who are unfit for surgery [4]. The endoscopic delivery of the drug further expands precision by improving the visibility of the engorged vessels and the dentate line [8], achieving low proportions of severe pain [12].

In parallel with these therapeutic advances, our group has explored the role of transperineal ultrasound (TPUS) for the pre- and postoperative assessment of HD. TPUS has emerged as a promising non-invasive imaging modality capable of visualizing hemorrhoidal cushions and vascular flow in real time and without compression [13], compared to endoanal ultrasound (EUS) [14, 15]. TPUS utility includes assessment of hemorrhoidal size and vascularization, identification of feeding vessels to support therapeutic planning, and follow-up monitoring to detect residual or recurrent disease without the need for invasive examination [13, 16]. Owing to its ease of use, wide availability, low cost, and applicability across pre-, intra-, and postoperative settings, TPUS represents a valuable tool for improving the understanding of HD pathophysiology [17].

Despite the increasing use of sclerotherapy, local hemodynamic changes induced by the procedure and their relationship with clinical outcomes have not yet been systematically investigated. A deeper understanding of these hemodynamic modifications may help clarify the mechanisms underlying treatment response or failure, which is particularly relevant given that sclerotherapy is a repeatable procedure and that non-responders or patients with treatment failure may ultimately require more invasive excisional interventions. In the present study, we assessed early local hemodynamic changes following endoscopic PF sclerotherapy using TPUS and analyzed their association with patient-reported outcome measures (PROMs).

## Materials and Methods

This is a prospective observational study and has been reported according to the Strengthening the Reporting of Observational Studies in Epidemiology (STROBE) guideline [18]. All procedures in studies involving human participants were performed by the ethical standards of the institutional and/or national research committee and with the 1964 Helsinki Declaration and its later amendments or comparable ethical standards. The study was approved by the Local Ethical Committee (IRCSS Istituto Oncologico “Gabriella Serio” Prot. 2146/CEL).

Included patients were those with HD who presented between June 2025 and October 2025 to the proctologic clinic of a district general hospital (Azienda Sanitaria Locale ASL Lecce, “Francesco Ferrari” Hospital, Casarano) and were treated with PF sclerotherapy. Indications for sclerotherapy consisted of symptomatic I–III-degree HD according to the Goligher classification [19, 20], as well as grade IV patients who, despite receiving thorough counselling about the limitations of sclerotherapy for this stage, declined hemorrhoidectomy or any other non-excisional procedure and specifically requested sclerotherapy. Excluded from the study were patients that did not attend both follow-up visits, those with associated anorectal pathologies (i.e., anal fistulas or abscesses, anal fissures, condylomas, tumors, rectocele, internal intussusception, rectal prolapse, ulcerative colitis, Crohn’s disease, or any other anoperineal disease), or that received previous anorectal surgery or hemorrhoidal procedures other than sclerotherapy.

### Initial Visit

Patients were initially assessed through a detailed history and a proctological evaluation, including perineal and digital rectal examinations. Symptoms were simultaneously scored using the Patient-Reported Outcome Measure–Hemorrhoidal Impact and Satisfaction Score (PROM-HISS) [21]. The PROM-HISS score consists of three domains: five HD symptoms (bleeding, pain, prolapse, soiling, itching; each scored from 1—less worrying—to 5—most worrying), impact of symptoms on daily activities (score 0—lowest—to 10—highest impact), and satisfaction with treatment (score 0—lowest—to 10—highest satisfaction) [21]. For the purpose of this study, the PROM-HISS instrument was applied in its validated form; however, only domains applicable across all study time points were included in the longitudinal analysis. The domain assessing impact on daily activities was excluded from comparative analysis because all patients reported minimal impairment at baseline (score 0–1 on a VAS scale), resulting in a floor effect that prevented meaningful comparison over time. The satisfaction domain was not included in the analysis because it is inherently post-treatment and therefore not applicable at baseline evaluation. Other data collected included basic demographics (age, sex), prior use of medical treatments (defined as flavonoids, oral products other than flavonoids, local creams, ointments), and Goligher grade (as reported by patients).

### TPUS

All TPUS examinations were performed during the initial visit using the ESAOTE MyLab XPRO80® (Genova, Italy) with an mC3-11 microConvex Array Transducer (3.0–11.0 MHz). Scans were conducted via a perineal approach, with the probe gently contacting the anal verge, as previously described [13, 16]. Minimal pressure was applied to optimize image acquisition without distorting the local anatomy. For each subject, three sonographic views were recorded: one aligned with the sagittal plane, one with the coronal plane, and a third along an oblique axis between them. Anatomical interpretation was based on criteria previously described in the literature [13, 16]. A color Doppler evaluation was also performed for each image by adjusting the volume of interest to include the internal anorectal region. Measurements of peak systolic velocity (PSV), end-diastolic velocity (EDV), and resistance index (RI) were obtained for each of the three assessed axes and recorded in a dedicated database.

### Endoscopic Sclerotherapy

**Fig. 1:**
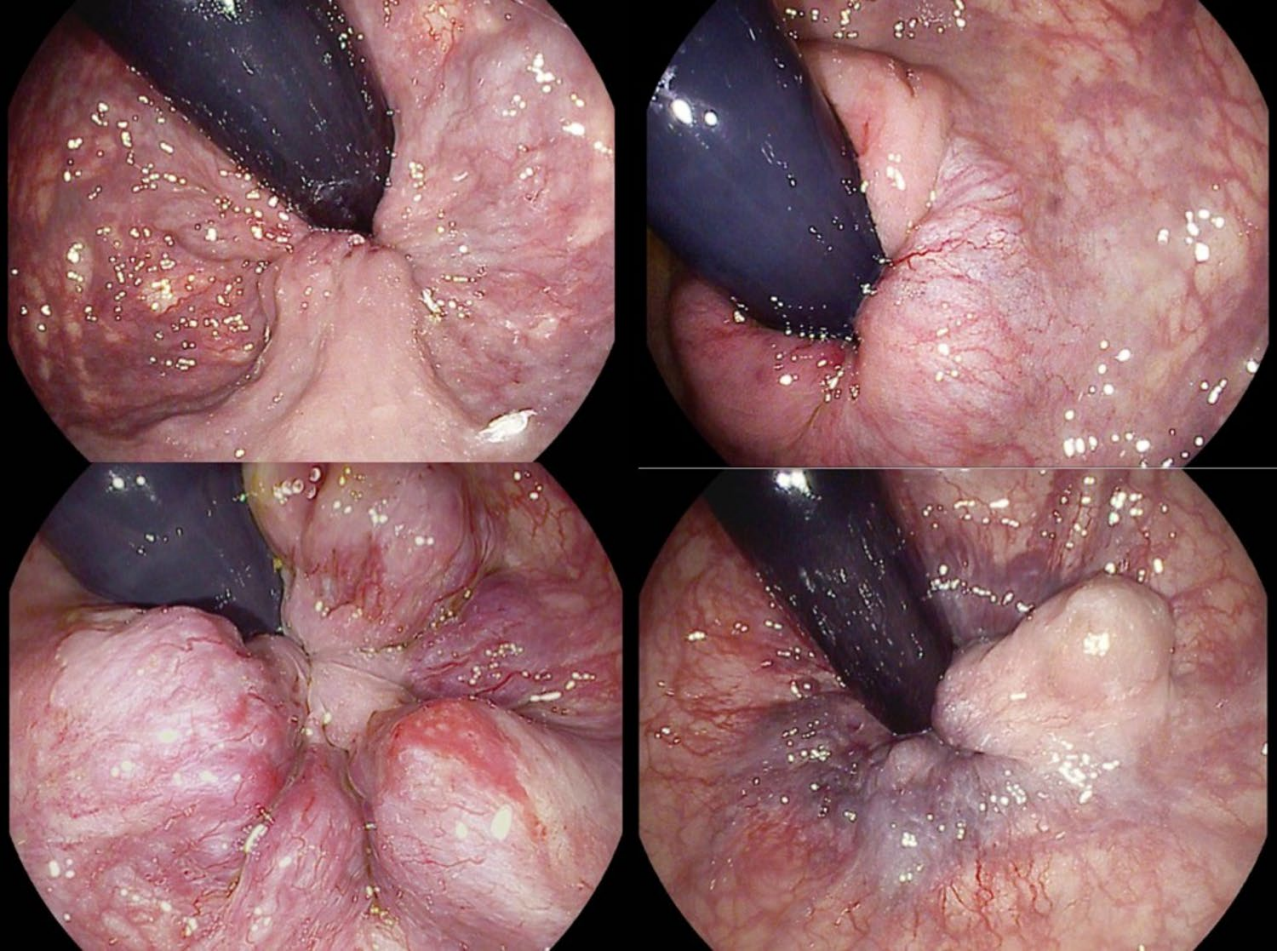
Representative retroflexed endoscopic views of hemorrhoidal disease

Patients meeting the inclusion criteria were treated during a separate appointment, performed in conjunction with a planned lower gastrointestinal endoscopic assessment. When bleeding or other symptoms suspicious for colorectal cancer were present, a complete colonoscopy or computed tomography colonography was requested, unless a recent examination was already available. In cases where a recent examination had been performed, a flexible sigmoidoscopy was nevertheless carried out to allow direct visualization of the internal hemorrhoids (Fig. 1) and to perform the sclerotherapy. Endoscopic sclerotherapy was therefore conducted at the conclusion of a diagnostic colonoscopy or sigmoidoscopy, once internal hemorrhoidal disease had been identified as the primary cause of bleeding.

**Fig. 2:**
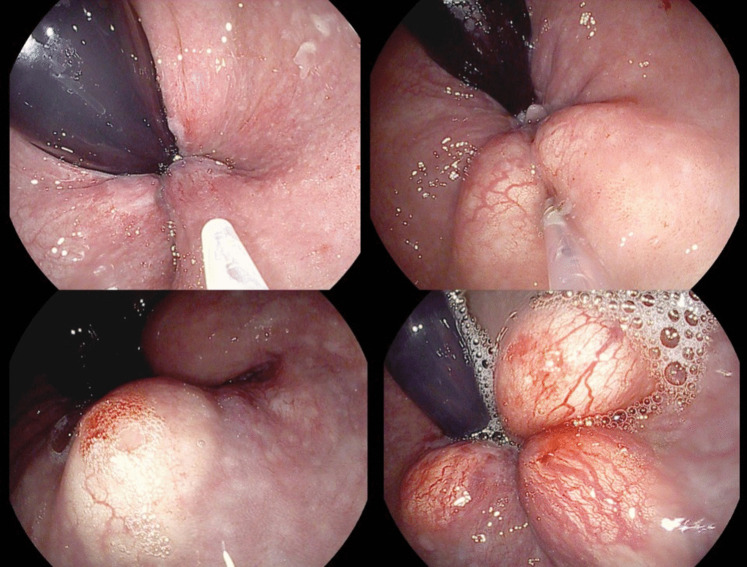
Endoscopic sclerotherapy procedure: positioning of the injection needle into the hemorrhoidal pile slightly above the dentate line (upper left); injection of polidocanol foam (upper right); formation of a characteristic white submucosal bleb (lower left); and final endoscopic appearance after treatment of all hemorrhoidal piles (lower right)

The procedures were performed using a Pentax EPK-i7010 processor and an E38-i10L colonoscope. With regard to sclerotherapy, patients were treated according to current consensus [2], in an outpatient setting and in the left lateral position. PF (Atoxysclerol® 3%, Gloria Med Pharma S.r.l., Menaggio, Italy) was prepared using the Tessari method [22], by mixing 2 mL of liquid polidocanol with 8 mL of air through two syringes connected by a three-way stopcock, obtaining a total volume of 10 mL of foam. Two milliliters of foam was injected into each hemorrhoidal pile during the retroflexed maneuver using a 23-G endoscopic single-use injection needle (Olympus, Hong Kong) (Fig. 2). All endoscopic injections, as well as clinical and TPUS assessments, were performed by a well-trained colorectal surgeon (GGr) with more than 5000 endoscopic procedures. TPUS images were further reviewed by a second Author (GGa), and discrepancies were resolved by discussion or consultation with a third author (VDS).

Following treatment, all patients were routinely followed up at 7 and 30 days in our outpatient clinic. During these visits, patients were asked about the presence of symptoms, the HISS score was calculated, and a TPUS assessment of PSV, EDV, and RI was conducted for each of the three anatomical planes examined (sagittal, coronal, and oblique), following the same protocol used at the baseline assessment.

### Outcomes

The primary outcome of this study was the longitudinal change in TPUS-derived hemodynamic parameters (PSV, EDV, and RI) from baseline to 7 and 30 days after endoscopic PF sclerotherapy. A subgroup analysis was performed according to disease severity, stratified into lower- (Goligher I–II) and higher-grade HD (Goligher III–IV), based on previously reported TPUS-derived hemodynamic patterns [13]. Secondary outcomes were to define the clinical outcomes and assess eventual correlations with the TPUS parameters.

### Power Analysis

The study aims to recruit at least 15 subjects in each group (lower- and higher-grade HD). The sample size was determined assuming a significance level (*α*) of 0.05 and a desired statistical power of 87. The power calculation was based on a two-sample one-sided *t*-test comparing group means for PSV (M1 = 8.3 vs. M2 = 10.5) with standard deviations (S1 = 2.9, S2 = 3.1), derived from the data previously published [13]. This analysis showed that 15 patients per group would provide a power of 87%.

### Statistical Analysis

All data were inserted into an Excel database (Microsoft, Redmond, Washington, USA) and analyzed with the Statistical Package for the Social Sciences Windows version 27.0 (SPSS, Chicago, Illinois, USA). Descriptive statistics used were the mean ± standard deviation for continuous parametric variables, the median and interquartile range (IQR) for continuous non-parametric variables, and frequencies for categorical variables. Normality assumptions were demonstrated with histograms and the Shapiro-Wilk test. Analysis of comparison between groups (higher vs. lower-grades HD) and over time (preoperative, 7th and 30th day follow-up) for PSV, ESV, RI, and HISS score was conducted with the Friedman test (continuous non-parametric variables), ANOVA one-way for age, and chi-square test for categorical variables (sex); Fisher’s exact test if the counts in cells were inferior to 5. *p* value less than 0.05 was considered statistically significant.

## Results

During the study period, 47 patients were initially assessed for eligibility. Six patients were not enrolled, as recruitment was discontinued before completion of baseline TPUS assessment and treatment. Of the remaining 41 patients who underwent baseline TPUS evaluation and PF sclerotherapy, 4 (9.8%) did not complete follow-up and were excluded from the final analysis. Complete follow-up data were therefore available for 37 patients, corresponding to 111 observations for each TPUS parameter across all time points (baseline, 7 days, and 30 days).

Descriptive statistics are reported in Table 1. The mean age of the study population was 60 ± 16 years; most patients were male (62.2%) and had received prior medical treatment before sclerotherapy (89.2%). No significant differences in baseline characteristics (age, sex, or previous medical treatment) were observed between lower- and higher-grade HD (Table 1). Preoperative PSV was 9.5 cm/s (IQR 7.0–12.8), and EDV was 2.2 (1.7–2.5), with no significant differences between groups (Friedman test, *p* = 0.843 and 0.069 respectively; Table 1). Preoperative RI was significantly higher in patients with lower-grade HD (Friedman test, *p* = 0.039), whereas the HISS score was significantly higher in patients with higher-grade HD (Friedman test, *p* = 0.020).

**Table 1:** Descriptive statistics of patients with hemorrhoidal disease stratified by Goligher classification. *HD* hemorrhoidal disease, *PSV* peak systolic velocity, *EDV* end diastolic velocity, *RI* resistance index

	Total (n = 37)	Low-grade HD (Goligher I–II) (*n* = 22)	High-grade HD (Goligher III–IV) (*n* = 15)	*p*
Age (years)	60 ± 16	59 ± 17	62 ± 13	0.931
Sex (males)	23 (62.2%)	13 (59.1%)	10 (66.7%)	0.738
Medical treatment prior to sclerotherapy	33 (89.2%)	19 (86.4%)	14 (93.3%)	0.633
PSV (cm/sec)				
• preop	9.5 (7.0–12.8)	9.9 (7.1–14.6)	9.5 (7.0–11.5)	0.843
• 7th day	8.0 (5.8–12.1)	7.8 (5.2–11.1)	8.6 (6.0–13.6)	0.164
• 30th day	8.3 (6.5–12.6)	8.1 (5.8–12.4)	9.3 (7.5–13.7)	**0.034**
EDV (cm/sec)				
• preop	2.2 (1.7–2.5)	2.2 (1.7–2.5)	2.2 (1.9–2.9)	0.069
• 7th day	2.2 (1.8–2.5)	2.1 (1.7–2.3)	2.3 (1.9–2.8)	**0.012**
• 30th day	2.2 (2.0–2.8)	2.2 (1.7–2.4)	2.7 (2.0–3.7)	** < 0.001**
Preop. RI				
• preop	0.77 (0.69–0.84)	0.78 (0.73–0.87)	0.74 (0.64–0.82)	**0.039**
• 7th day	0.73 (0.64–0.80)	0.73 (0.65–0.79)	0.72 (0.61–0.80)	0.620
• 30th day	0.72 (0.64–0.80)	0.73 (0.67–0.82)	0.72 (0.62–0.79)	0.085
HISS score				
• preop	11 (9–12.5)	10 (8–11)	11 (10–14)	**0.020**
• 7th day	7 (6–7)	6 (5–7)	7 (7–8)	**0.001**
• 30th day	7 (5–8.5)	6 (5–8)	8 (7–9)	**0.001**

When considering the entire cohort, PSV showed a significant reduction at 7 days compared with baseline (Friedman test, *p* = 0.008), while the difference was no longer significant at 30 days (*p* = 0.052). In patients with lower-grade HD, PSV values were significantly reduced at both 7 days (*p* = 0.005) and 30 days (*p* = 0.007) compared with preoperative baseline. In contrast, no significant changes in PSD were observed in patients with higher-grade HD at either 7 days (*p* = 0.391) or 30 days (*p* = 0.767).

Regarding EDV, no significant changes were observed at 7 or 30 days compared with baseline in the overall cohort (*p* = 0.334 and *p* = 0.172, respectively) or in patients with lower-grade HD (*p* = 0.383 and *p* = 0.794, respectively). Conversely, patients with higher-grade HD showed a significant increase in EDV at 30 days compared with baseline (*p* = 0.012), but not at 7 days (*p* = 0.564). Finally, when analyzing the entire cohort, RI showed a significant reduction at both 7 and 30 days compared with baseline (Friedman test, *p* = 0.002 and *p* = 0.001, respectively). Similar findings were observed in patients with lower-grade HD (*p* = 0.002 and *p* = 0.005, respectively). In contrast, no significant changes in RI were observed in patients with higher-grade HD at either follow-up time point (*p* = 0.073 at 7 days and *p* = 0.315 at 30 days; Table 1).

HISS score significantly decreased over time in the entire cohort, with lower values at both 7 and 30 days compared with baseline (Friedman test, *p* < 0.001 for both comparisons). Similar significant reductions were observed in patients with lower-grade HD (*p* < 0.001 at both time points) and in those with higher-grade HD (*p* = 0.001). However, in patients with higher-grade HD, HISS values significantly increased between 7 and 30 days post-treatment (*p* = 0.027).

## Discussions

**Fig. 3:**
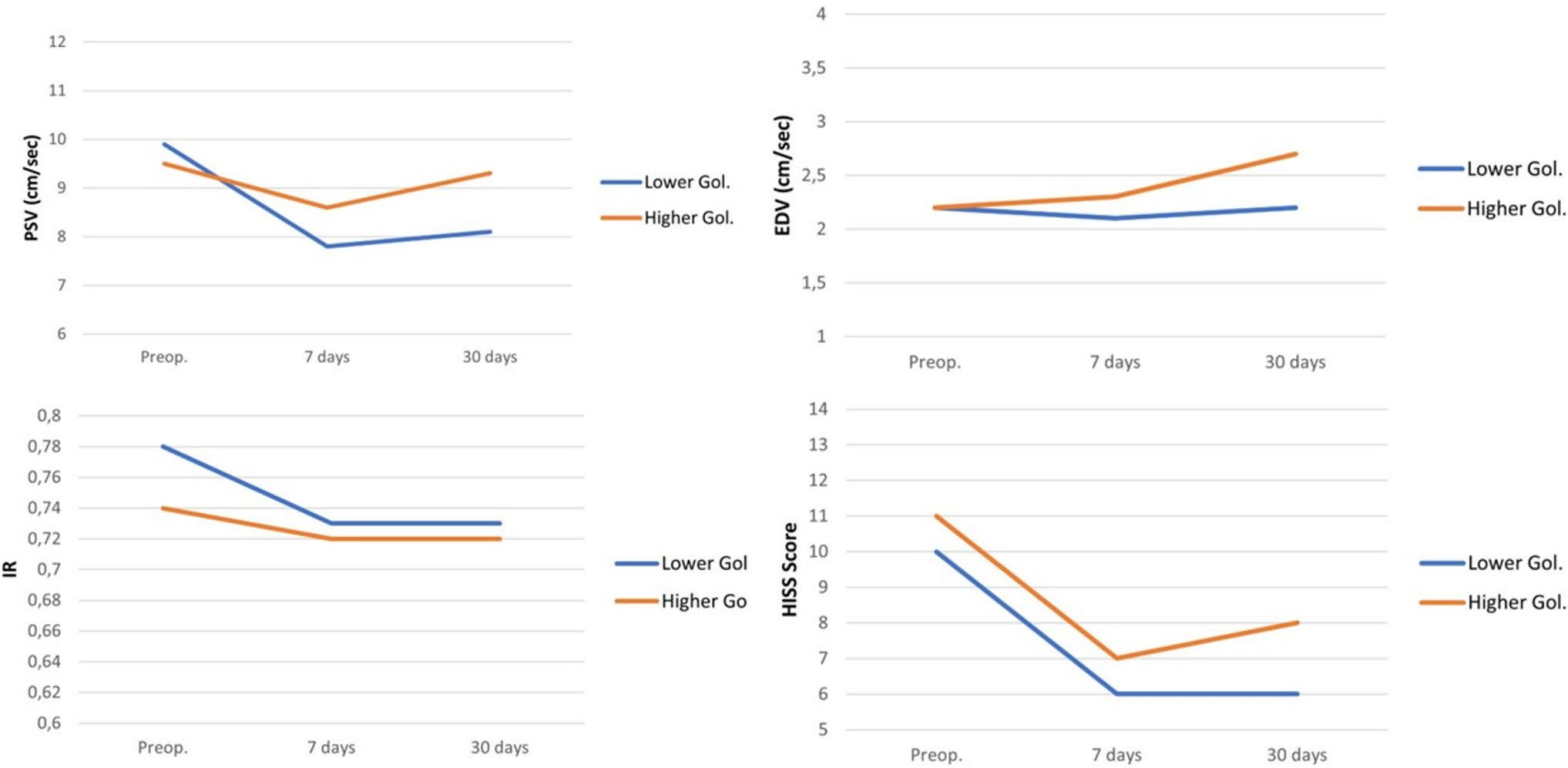
Median peak systolic velocity (PSV), end diastolic velocity (EDV), resistance index (RI), and HISS score values in patients with lower- and higher-grade Goligher hemorrhoidal disease at baseline, 7 days, and 30 days of follow-up

This prospective observational study provides novel insights into the early hemodynamic changes induced by endoscopic PF sclerotherapy in HD patients and explores their relationship with PROMs; to our knowledge, it is among the first to systematically evaluate Doppler-derived parameters following sclerotherapy and to correlate these objective vascular changes with symptom evolution. Similarly, to previous TPUS findings [13], no significant differences in preoperative PSV and EVD were observed between patients with lower- and higher-grade HD. After treatment, however, the two groups showed divergent hemodynamic responses (Fig. 3). Patients with lower-grade HD exhibited significant reductions in both PSV and RI at 7 and 30 days after sclerotherapy, indicating a short-term modification of local hemorrhoidal arterial inflow. In contrast, no significant variations in PSV or RI were observed in patients with higher-grade HD. In this setting, the divergent post-treatment hemodynamic response observed between groups is unlikely to be explained by baseline vascular differences, but rather suggests a grade-dependent capacity for vascular remodeling and consequent hemodynamic response after PF sclerotherapy.

From a Doppler ultrasound perspective, these findings support a grade-dependent hemodynamic response to PF sclerotherapy. PSV reflects arterial inflow to the hemorrhoidal plexus, while EDV is mainly influenced by distal vascular resistance and venous outflow; RI represents a composite index of vascular resistance within the explored district. The combined reduction in PSV and RI observed in lower-grade HD suggests effective modulation of arterial inflow with functional remodelling of the hemorrhoidal vascular bed. These observations are consistent with the proposed mechanism of action of sclerotherapy, which aims to induce endothelial damage, fibrosis, and functional reduction of hemorrhoidal vascular supply [23]. Conversely, the absence of significant changes in PSV and RI, along with a late increase in EDV in higher-grade HD, may reflect persistent venous congestion and altered venous outflow dynamics. This finding could be compatible with the recruitment of alternative drainage pathways; however, this interpretation remains speculative, as no direct visualization of collateral circulation (e.g., venography or angiographic correlation) was available in the present study.

The consistent and significant reduction in HISS scores across the entire cohort confirms the clinical effectiveness of PF sclerotherapy in alleviating HD-related symptoms, in line with previous reports [5, 9]. Notably, symptomatic improvement was observed even in the absence of marked Doppler changes in higher-grade HD, suggesting that mechanisms other than pure flow reduction—such as decreased bleeding from superficial mucosal vessels or reduced local inflammation—may contribute to early clinical benefit. However, the subsequent increase in symptom burden between 7 and 30 days in this subgroup suggests that clinical improvement may be transient if not supported by durable vascular remodeling. This dissociation between early symptomatic improvement and objective hemodynamic response may help explain the higher recurrence rates reported after sclerotherapy in advanced HD [24] and reinforce the concept of PF sclerotherapy as a palliative or symptomatic option in Goligher III/IV patients unfit for, or unwilling to undergo, surgery. From a clinical perspective, the assessment of early hemodynamic changes may provide a functional biomarker of treatment response, allowing the identification of responders and non-responders shortly after the procedure. This may be particularly relevant for tailoring follow-up strategies and guiding early retreatment decisions. In lower-grade hemorrhoidal disease, the observed reduction in arterial inflow supports a disease-modifying effect of PF sclerotherapy, whereas in higher-grade disease, the absence of significant hemodynamic changes despite transient symptom improvement may help identify patients at risk of early relapse, who could benefit from closer monitoring, retreatment, or alternative therapeutic strategies.

Our group has recently expanded the application of TPUS in proctology, employing this technique for both pre- and postoperative assessment of patients with HD [13, 16]. Its ease of learning, widespread availability, and lower costs compared with dedicated EUS probes make this technique particularly useful, especially when applied to a highly prevalent condition such as HD [25]. In the preoperative setting, TPUS was shown to allow a functional stratification of HD severity, distinguishing a lower-grade HD (Goligher I–II), characterized by clinical symptoms without significant hemodynamic alterations compared with controls, from a higher-grade HD (Goligher III–IV), in which symptoms are associated with increased peak systolic velocity PSV [13]. In the postoperative setting, TPUS has also proven useful as a screening tool in symptomatic patients after hemorrhoidectomy, where normal findings were associated with a high negative predictive value for recurrent disease [16]. From a methodological standpoint, the current study supports the feasibility and reproducibility of TPUS as a non-invasive tool for functional assessment of HD. Compared with conventional proctological examination, TPUS offers the advantage of objective quantification of vascular parameters (PSV, EDV, RI) and the possibility of longitudinal follow-up without patient discomfort. Furthermore, the ability to detect early hemodynamic changes may be particularly valuable in identifying suboptimal responders, tailoring follow-up strategies, and potentially guiding retreatment decisions. However, external assessment of vascular flow appears more physiological than EUS, as local vessel compression by the EUS probe may interfere with Doppler measurements [14, 15]. While TPUS appears to be a promising and informative research tool, its routine implementation in everyday clinical practice remains to be established. At present, symptom assessment and physical examination continue to represent the cornerstone of patient management, and TPUS incremental value requires further validation in larger, prospective studies.

Some limitations should be acknowledged. First, the sample size was relatively small, particularly when stratified by Goligher grade, which may have limited the statistical power to detect subtler differences, especially in the higher-grade group. Second, the inclusion of patients with higher-grade disease (Goligher III–IV), although clinically justified based on patient preference after counselling, may have influenced the interpretation of the results. In particular, the different underlying pathophysiology and expected response to sclerotherapy in advanced disease may have introduced heterogeneity in both hemodynamic and clinical outcomes. Third, the follow-up period was short and focused on early outcomes; longer-term TPUS assessments are necessary to determine whether early hemodynamic patterns can predict recurrence or sustained response. Fourth, comparison with previously published studies investigating post-treatment hemodynamic changes in HD is not feasible, as those studies employed different imaging modalities (EUS versus TPUS) and different therapeutic approaches (transanal hemorrhoidal dearterialization versus endoscopic sclerotherapy) [14, 15]. Direct comparative studies using the same assessment method across different treatment strategies would be required to allow meaningful comparisons and would be of particular interest to further elucidate treatment-specific hemodynamic effects.

## Conclusions

PF sclerotherapy induces significant early hemodynamic changes detectable by TPUS in patients with lower-grade HD, and these changes parallel a short-term clinical improvement. Conversely, in higher-grade HD, no significant hemodynamic modifications were observed, and the initial symptomatic benefit tended to diminish over time. These findings support the role of sclerotherapy primarily as a palliative strategy in advanced disease, particularly in patients who are unfit for, or unwilling to undergo, hemorrhoidectomy. TPUS further emerges as a promising adjunctive tool for functional assessment and postoperative follow-up, with potential implications for patient selection, treatment stratification, and outcome prediction.

## Data Availability

No datasets were generated or analysed during the current study.
